# Ferroptosis and protein translation: emerging perspectives in the research of myocardial infraction

**DOI:** 10.3389/fcvm.2025.1592333

**Published:** 2025-05-02

**Authors:** Qi Lan, Qiu-Yu Liu, Wei-Cai Qiu, Ling-Ling Liang, Zhen-Xun Wan, Ting Peng, Ping Liu, Gang Luo, Ming-Tai Chen, Meng-Nan Liu

**Affiliations:** ^1^ Department of Cardiovascular Medicine, Affiliated Traditional Chinese Medicine Hospital, Southwest Medical University, Luzhou, China; ^2^ School of Pharmacy, Southwest Medical University, Luzhou, China; ^3^ Department of Cardiovascular Disease, Shenzhen Traditional Chinese Medicine Hospital, Shenzhen, China

**Keywords:** myocardial infarction, ferroptosis, protein translation, mechanism, cardiovascular disease

## Abstract

Myocardial infarction, as the principal type of ischemic heart disease, has currently become the focus of research on its prevention and treatment strategies. From the perspective of myocardial infarction pathogenesis, it is urgent to impede the progression of this disease and improve diagnosis and treatment techniques. Ferroptosis, a form of programmed cell death mechanistically distinct from apoptosis and autophagy, is implicated throughout the pathogenesis of myocardial infarction. Dysregulation of protein translation leads to abnormal protein expression, disruption of cellular signaling, and cell dysfunction, thereby disturbing normal cellular function and exacerbating disease progression. Consequently, clarifying the mechanism of protein translation dysregulation in ferroptosis during myocardial infarction will enhance the understanding of the pathogenesis of myocardial infarction. In this review, the latest research progress in the relationship between protein translation and ferroptosis is collected. The mechanisms by which they regulate myocardial infarction are explored, and the current research status of the role of protein translation in different stages of ferroptosis is introduced. These findings are expected to provide valuable insights for clarifying the pathophysiological mechanisms of myocardial infarction and for precise treatment.

## 1 Introduction

Myocardial infarction (MI), as an acute condition within ischemic heart disease, is the leading cause of global mortality, affecting approximately 32% of the population and resulting in an estimated 17.9 million deaths annually (1). The death of myocardial cells characterizes MI due to prolonged ischemia and hypoxia, which activates pathophysiological processes such as inflammatory responses, oxidative stress, and other cellular and intercellular reactions. These processes lead to myocardial cell hypertrophy, myocardial interstitial fibrosis, cell apoptosis and even ferroptosis (2–5). Given that mature cardiomyocytes cannot regenerate, the damaged cardiac tissue is progressively replaced by fibrotic scar tissue, culminating in cardiac remodelling and heart failure (6). Despite advancements in the care and management of MI patients, current therapeutic strategies primarily focus on the early and timely restoration of ischemic myocardial reperfusion. However, effective treatments for the pathological changes associated with infarction remain elusive, and long-term prognosis continues to be unfavourable (7). Consequently, it is imperative to delay the progression of MI by elucidating its molecular mechanisms and to enhance diagnostic and therapeutic technologies.

The occurrence and progression of diseases are driven by dysregulated translation, which leads to abnormal protein expression, disrupted cellular signaling and dysfunctional cellular processes. Mutations or modifications of mrna, trna, translation factors, ribosomes or regulatory elements that are dysregulated at the expression level during the four phases of protein translation, namely initiation, elongation, termination, and recycling, causing aberrant protein expression, which disrupts normal cellular processes and exacerbates the onset and progression of disease, and the key to the development of cardioprotective strategies is an understanding of the pathological process of cardiomyocyte injury (8–11). Ferroptosis, an emerging form of iron-dependent cell death in the pathogenesis of MI, is primarily characterized by lipid peroxidation, with its key biochemical features including increased levels of reactive oxygen species (ROS), Fe^2+^ and malondialdehyde (MDA), as well as decreased levels of antioxidant enzymes such as glutathione peroxidase 4 (GPX4) (12–14). Cardiomyocytes, which contain a high proportion of unsaturated fatty acids, are more susceptible to ferroptosis due to the ease with which these fatty acids are oxidized into lipid peroxides (15). Previous studies have demonstrated that ferroptosis inhibitors or iron chelators significantly ameliorate MI (16, 17). However, the molecular mechanisms of ferroptosis, including the specific components of damaged cell membranes, remain largely unknown. Therefore, targeting the molecular mechanisms of ferroptosis to inhibit the initiation and progression of MI may represent a critical cardioprotective strategy.

In recent years, ferroptosis has been extensively studied in the context of cardiovascular diseases (cvds) and has emerged as a significant focus in MI research. Understanding the regulation of ferroptosis in MI is considered a prerequisite for reducing cardiomyocyte death and its associated morbidity and mortality. The role of protein translation in the mechanisms of ferroptosis has gradually become a rising area of interest within ferroptosis research. Elucidating the mechanisms by which dysregulated protein translation contributes to ferroptosis in MI is expected to provide deeper insights into the pathogenesis of MI and guide the development of novel therapeutic strategies (18). This review summarises the latest understanding of the relationship between protein translation and ferroptosis, and the mechanisms by which they regulate MI are discussed. Furthermore, the current status of experimental and clinical research on the different stages of protein translation in ferroptosis is described, to offer emerging perspectives on ferroptosis research in MI and references for precision medicine in the treatment of this condition.

## 2 Overview of ferroptosis

**Figure 1:**
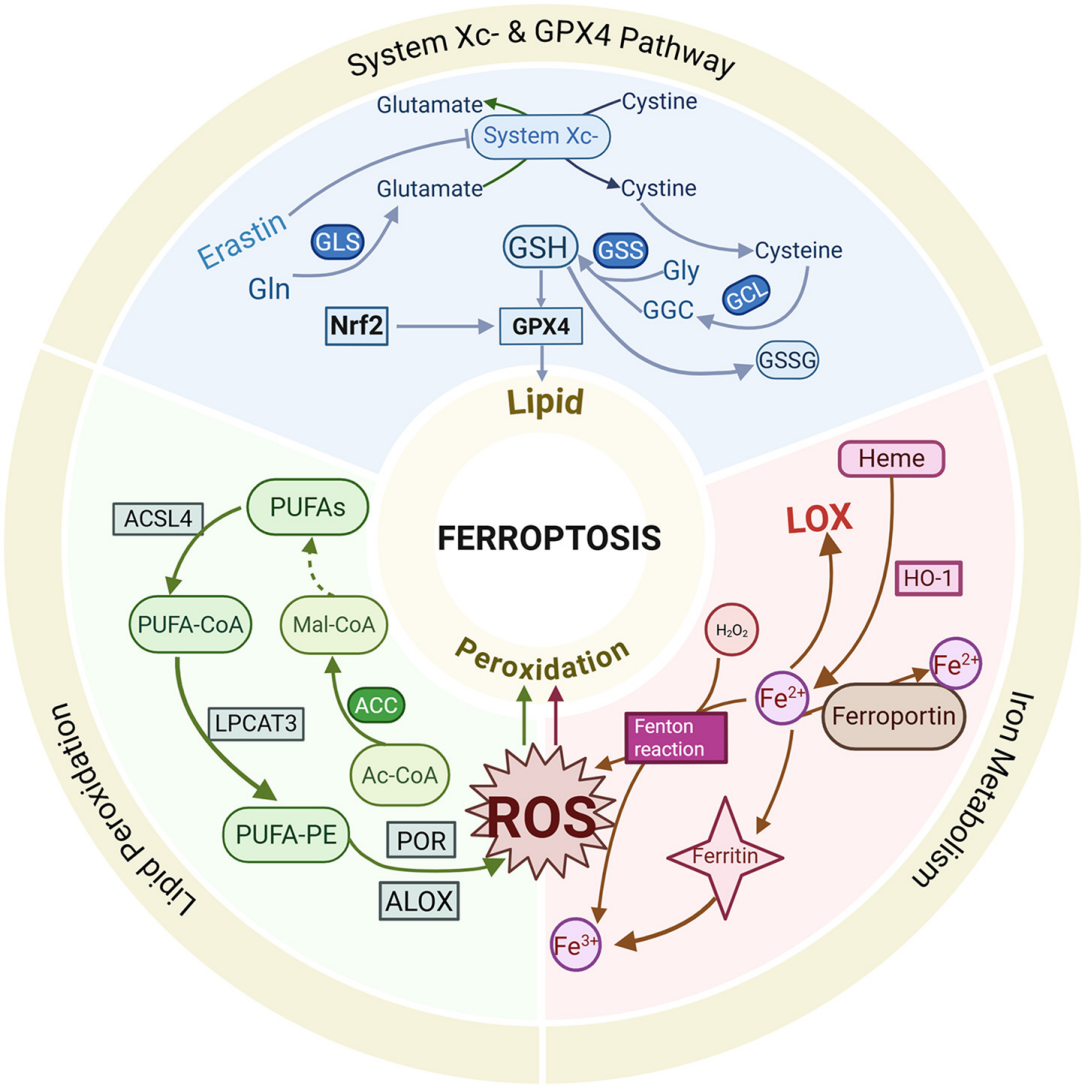
Brief mechanism of ferroptosis.

Ferroptosis, a novel mode of cell death induced by iron accumulation and lipid peroxidation, was first proposed by Dixon in 2012 (19). The core of ferroptosis lies in the influence of iron inducers on GPX levels through multiple pathways, leading to elevated lipid ROS and reduced antioxidant capacity, ultimately resulting in oxidative stress and cell death (20). The primary molecular mechanisms of ferroptosis include the inactivation of GPX4, dysregulation of iron metabolism and lipid peroxidation. As is shown in Figure 1.

### 2.1 System X^c−^ and GPX 4

System X^c−^ is a ubiquitous cystine (Cys)/glutamate (Glu) antiporter composed of light and heavy chains encoded by SLC7A11 and SLC3A2 (21). It facilitates the synthesis of reduced glutathione (GSH) by mediating the transmembrane exchange of Cys and Glu (22). Cys is imported into cells via the action of system X^c−^, while GSH synthesis is supported by glyoxylate carbo ligase (GCL) enzymes and glutathione synthetase (GSS) (23). At the same time, GSH acts as a co-substrate for GPX4 between the reduced and oxidised states (24). Inhibition of system X^c−^ or inactivation of GPX4 leads to ROS accumulation and ferroptosis induction (25, 26). Suppression of system Xc- results in decreased GSH levels, triggering oxidative damage and ferroptosis (27). GSH, a tripeptide composed of Cys, Glu and glycine, has been shown to exert antioxidant functions by binding to free radicals, heavy metals, etc (28). System X^c−^ affects GSH synthesis by regulating extracellular Glu levels (29). Studies have shown that inhibiting glutamine metabolism and synthesis reduces Glu production, thereby attenuating the ferroptosis pathway and alleviating ischemia-reperfusion-induced myocardial injury (30). Ferroptosis inducers, such as Erastin, extracellular Glu, sorafenib and sulfasalazine, have been reported to induce ferroptosis by blocking system X^c−^'s function by affecting Glu uptake and GSH synthesis (31–34).

Selenoprotein GPX4 is a critical peroxide degradation enzyme that utilizes GSH to generate glutathione disulfide (GSSG) and convert toxic lipid peroxides into alcohols, thereby preventing lipid peroxidation and maintaining cellular redox homeostasis (35). GSH serves as a key regulator of GPX4, and inhibition of system X^c−^ leads to GSH depletion and GPX4 inactivation, impairing cellular antioxidant capacity and increasing the risk of lipid peroxidation, which promotes the occurrence of ferroptosis (25). Genetic ablation of GPX4 or indirect suppression of GPX4 through upregulation of activating transcription factor 3 (ATF3) by Erastin results in the accumulation of lipid peroxides, subsequently triggering ferroptosis (27). Ding et al. Were the first to reveal a novel pathway for inducing ferroptosis through GPX4 ubiquitination and demonstrated that the anti-TNBC effects of DMOCPTL are primarily induced by GPX4 ubiquitination, which is achieved by direct binding to the GPX4 protein, thereby leading to ferroptosis and apoptosis (36). Currently, GPX4 is regarded as a crucial target for triggering ferroptosis.

### 2.2 Intracellular iron metabolism

Iron is naturally present in the human body, and its redox activity promotes ROS production and lipid peroxidation, ultimately leading to ferroptosis (37). Iron metabolism plays a pivotal role in the mechanisms of ferroptosis, with iron overload being one of the primary hallmarks driving this process (38). The normal physiological functions of iron in the human body are contingent on its uptake and excretion, which are regulated by transferrin receptors and ferroportin. Iron accumulation is positively correlated with intracellular iron levels, and excess redox-active iron generates ROS through the Fenton reaction, catalyzing the production of lipid peroxides and resulting in radical-mediated damage that induces ferroptosis (39). Ferritin, an intracellular iron storage protein, oxidises ferrous iron (Fe^2+^) to ferric iron (Fe^3+^), thereby preventing the Fenton reaction and subsequent oxidative damage (40). However, ferritin degradation leads to the release of stored iron and induces ferroptosis. Heme oxygenase-1 (HO-1), which degrades heme to release Fe^2+^, is involved in maintaining iron homeostasis, and its overexpression increases free iron levels, thereby contributing to the accumulation of lipid peroxides, leading to ferroptosis (41). The sensitivity to erastin-induced ferroptosis is influenced by alterations in the transcription of pivotal iron-regulating genes (31). Heat shock protein beta-1 (HSPB1), a critical gene in ferroptosis, impedes ferroptosis progression by downregulating intracellular iron levels by reducing the expression of telomeric RNA-binding factor 1 (TRF1) and inhibiting its expression suppresses Erastin-induced ferroptosis (42).

### 2.3 Lipid peroxidation and Its induction mechanisms

Lipid peroxidation plays a critical role in the mechanisms of ferroptosis, and its induction is mediated through non-enzymatic reactions (e.g., the Fenton reaction) and enzymatic reactions [e.g., lipoxygenase (LOX)]. The activity of non-heme, iron-containing enzymatic effectors such as LOX influences the accumulation of lipid peroxides, thereby promoting ferroptosis. LOX, pivotal in lipid peroxidation, has been shown to mitigate Erastin-induced ferroptosis damage when its activity is reduced (43). The complex formed by the binding of LOX to phosphatidylethanolamine-binding protein 1 (PEBP1) regulates the process of lipid peroxidation, initiating the ferroptosis program (44). Fe^2+^ and LOX jointly act on polyunsaturated fatty acids (PUFA), producing lipid peroxides that disrupt membrane structure and function (45). Pufas, such as arachidonic acid (AA) and adrenic acid, are essential components of phospholipids and preferred substrates for LOX, and their abundance and localization directly influence lipid peroxidation and cellular susceptibility to ferroptosis (46). The formation of ROS on lipids is mediated by hydrogen peroxide. Pufas are incorporated into membrane phospholipid (PL) through the combined action of acyl coenzyme A synthetase long-chain family member 4 (ACSL4) and lysophosphatidylcholine acyltransferase 3 (LPCAT3) (47). Acyl coenzyme A synthetase long-chain family member 3 (ACSL3) activates monounsaturated fatty acid (MUFA), competing with PUFA for PL integration. The oxidation of pufas alters membrane structure and fluidity, increasing membrane permeability and reducing membrane integrity. This membrane instability may lead to the formation of pores and micelles, ultimately triggering ferroptosis (48). Protein kinase C-mediated phosphorylation of HSPB1 has also been found to inhibit lipid ROS accumulation, affecting ferroptosis (42).

During ferroptosis, fatty acid metabolism genes, including ACSL4 and LPCAT3, regulate the insertion of PUFA into the cell membrane (49). The upregulation of ACSL4 expression enhances cellular susceptibility to ferroptosis, whereas its silencing effectively inhibits this pathological process (50). Accumulation of lipid ROS triggers ferroptosis when the endogenous antioxidant system of the cell is imbalanced. Deletion of ACSL4 and LPCAT3 renders cellular resistance to ferroptosis, highlighting their pivotal regulatory roles in ferroptosis. In addition, deficiency in coenzyme Q10, an antioxidant, is associated with increased ferroptosis sensitivity (51). The transcription factor NF-E2-related factor-2 (Nrf2) emerges as a critical molecular determinant in ferroptosis regulation through its capacity to prevent lipid peroxide accumulation and maintain cellular viability. Although Kelch and ECH-associated protein 1 (KEAP1) activates Nrf2 to exert an anti-ferroptosis effect, numerous bioactive lipids paradoxically inhibit Nrf2 functionality, thereby playing essential roles in ferroptosis cascade signaling (52). The enzymatic activity of GPX4, a key antioxidant enzyme, exerts regulatory control over lipid peroxidation and ferroptosis progression. Pharmacological inhibition of GPX4 elevates ROS levels, subsequently promoting ferroptosis (53). Collectively, lipid peroxidation is established to play a central role in ferroptosis pathogenesis, though the underlying induction mechanisms exhibit complex and multifaceted characteristics that warrant further investigation to elucidate precise regulatory pathways.

## 3 Protein translation in ferroptosis

### 3.1 Overview of protein translation

The process of protein translation is the final step of the central law of molecular biology, and its translation process can be divided into four distinct phases: translation initiation, elongation, termination and recycling. In the initiation phase, translation initiation factors orchestrate mRNA binding to the ribosomal subunits, facilitate the recognition of the start codon, and align it with fMet-tRNAMet in the ribosomal P site (54). Dysregulation of the highly regulated translation initiation correlates with the diseased cellular state. Dysregulation of protein expression and protein mutations associated with post-translational modifications often suppress translation initiation. The elongation phase is a core step in protein translation and mainly includes codon recognition of aminoacyl tRNAs, peptide bond formation and translocation (55). Dysregulation of elongation significantly affects translation efficiency and fidelity, potentially leading to translation errors and protein misfolding, which may subsequently trigger cellular dysfunction and disease pathogenesis. Therefore, investigation into the mechanism of elongation dysregulation is essential for understanding disease procession and identifying potential therapeutic targets. During termination, when the termination codon (e.g., UAG, UGA, or UAA) of an mRNA enters the ribosomal A site, the protein release factor complex eRF1/eRF3-GTP binds to the A site and induces the termination of protein synthesis (56). Dysregulation of termination may produce truncated or aberrant proteins, disrupting proteostasis and affecting cellular function. Finally, during the ribosome recycling phase, the large (mt-LSU) and small (mt-SSU) ribosomal subunits are separated, and the mRNAs are released in preparation for a new translation cycle (57).

### 3.2 Mechanisms of protein translation in ferroptosis

The activation of ferroptosis is governed by the dynamic equilibrium between pro-ferroptotic factors and cellular defence systems. Protein synthesis pathways exhibit dual roles in ferroptosis regulation, with their directionality dictated by the cellular microenvironment. The rate-limiting step of protein synthesis is translation initiation, a process finely modulated by a family of proteins termed eukaryotic initiation factor (EIF) (58). Studies have demonstrated that phosphorylation of eIF2α reduces cellular sensitivity to peroxidation by suppressing protein synthesis, thereby exerting anti-ferroptotic effects (59). Previous studies have shown that the PERK/eIF2α axis of integrated stress response (ISR) is involved in the process of inhibiting protein synthesis during cardiac ischemia/reperfusion, and selectively targets mitochondrial complex components during cardiac reperfusion to further weaken protein translation and reduce the production of related ROS (60). Cellular susceptibility to ferroptosis is primarily determined by intracellular iron homeostasis, antioxidant capacity, and peroxidation levels. Excessive ROS production, particularly oxidative stress triggered by GSH depletion, is critical for ferroptosis (61). The mammalian target of rapamycin (mTOR) signaling pathway plays a pivotal role in cellular stress responses and ferroptosis regulation through its control over the assembly and functionality of translation initiation complexes (62).

Mitochondria, recognized as the energy metabolism hubs of eukaryotic cells, possess a protein translation system endowed with unique regulatory significance in ferroptosis control. Mitochondrial ribosomes (MR) are responsible for the expression of genes encoded by mitochondrial RNA (mtRNA), while mitochondrial translation factors such as mt-IF2 modulate cellular fate through the regulation of mitochondrial ROS levels (39). Noncoding RNAs (ncRNAs) play critical roles in ferroptosis regulatory networks: miRNAs interfere with Glu metabolism by modulating TCA cycle and respiratory chain functions, thereby reducing ROS generation; miR-7-5p targets transferrin to diminish iron uptake; and pro-ferroptotic miRNAs directly engage the SLC7A11/GPX4 system to accelerate lipid peroxidation (63, 64). For instance, miR-485 has been demonstrated to influence iron metabolism by targeting hepcidin, with its upregulation potentiating ferroptosis induction (65).

Post-translational modification (PTM) represents another critical layer in regulating ferroptosis. Ubiquitination, phosphorylation, acetylation, and methylation, among other PTMs, precisely modulate cellular sensitivity to iron-dependent oxidative stress by altering protein stability, functional localization, and interaction networks. Ubiquitination is a highly conserved PTM whose reversal process is mediated by deubiquitinase (DUB) (66). Histone phosphorylation is involved in DNA damage repair, while non-histone acetylation regulates transcriptional activity, protein stability and subcellular localization (67, 68). Methylation modifications predominantly occur on the side chains of arginine (Arg) and lysine (Lys) residues (69, 70). N6-methyladenosine (m6A), the most prevalent RNA modification, plays a key role in regulating programmed cell death through the coordinated actions of methyltransferases, reader proteins, and demethylases (71). The dynamic changes in m6A modification are closely associated with disease progression and pathogenesis.

## 4 Critical role of ferroptosis in myocardial infarction

**Figure 2:**
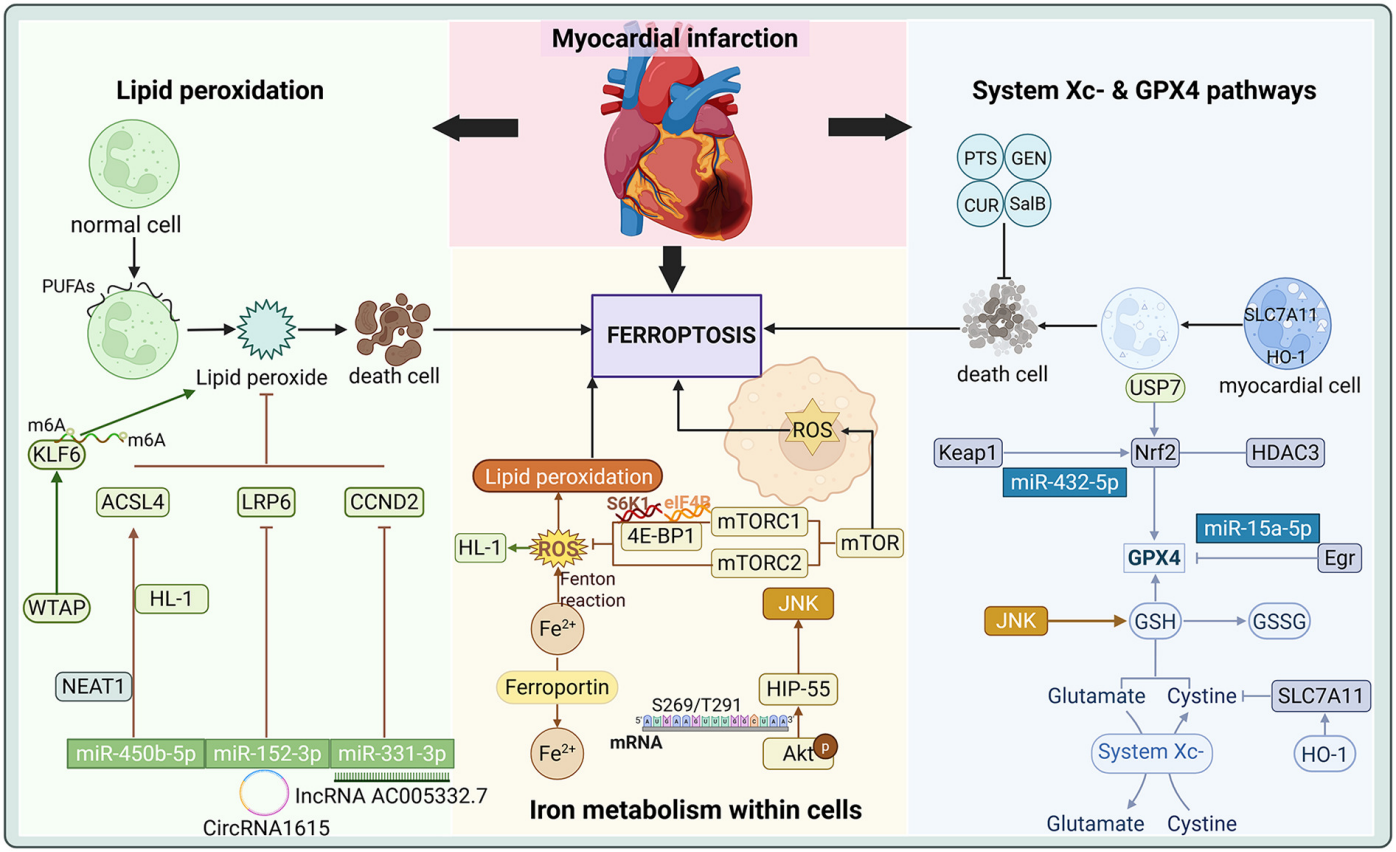
Critical Role of Ferroptosis in Myocardial Infarction.

Recent studies have demonstrated that protein translation is crucial in regulating ferroptosis. MI, resulting from coronary artery occlusion, triggers ferritin degradation, leading to iron overload, ROS accumulation, and iron-catalyzed lipid peroxidation in the heart (72, 73). These processes induce ferroptosis, significantly impacting the survival and death of cardiomyocytes. Below is a detailed exploration of the regulatory mechanisms of ferroptosis in MI based on three significant aspects of current research. As is shown in Figure 2 and Table 1.

**Table 1:** Translation of ferroptosis related proteins in myocardial infarction.

Regulatory mechanisms	Key molecules	Significance in myocardial infarction	Related research			Reference
			Efficacy	Type of study	Experimental model	
System Xc- and GPX4 Pathways	GPX4	Downregulation of GPX4 promotes cardiomyocyte ferroptosis	miR-15a-5p directly targets GPX4 to promote ferroptosis	*in vivo*	Rats with ligation of left anterior descending coronary artery	(74)
			Curdione increases the expression of GSH and GPX4 and disrupts the interaction between Keap1 and Trx1 in the Keap1/Trx1/GPX4 pathway, thereby alleviating myocardial infarction	*in vivo*	Mouse induced by subcutaneous injection of ISO	(17)
				*in vitro*	Rat H9c2 cells cultured by ISO	
			Geniposide upregulates the expression of Grsf1 in GPX4, thereby reducing iron overload in myocardial infarction	*in vivo*	Rats with ligation of left anterior descending coronary artery	(75)
				*in vitro*	H_2_O_2_-induced Primary cardiomyocytes and Rat cardiac H9c2 cells	
	Nrf2	Upregulation of Nrf2 expression inhibits ferroptosis in myocardial infarction	miR-432-5p enhances antioxidant capacity by activating Nrf2	*in vivo*	Rats with ligation of left anterior descending coronary artery	(76)
				*in vitro*	Oxygen Deprivation and Reoxygenation Model of primary cardiomyocytes	
			Salvianolic acid B inhibits ferroptosis by upregulating the expression of Nrf2 in the Nrf2/GPX4 axis	*in vivo*	Rats with ligation of left anterior descending coronary artery	(77)
			Panaxatriol saponin inhibit oxidative stress and alleviate ferroptosis in myocardial infarction by blocking the Nrf2 binding site in Keap1	*in vivo*	Rats with ligation of left anterior descending coronary artery	(78)
				*in vitro*	Rats CFs	
			AKR1C3 activates the Keap1/Nrf2/ARE pathway to mitigate ferroptosis following myocardial infarction	*in vivo*	Rats with ligation of left anterior descending coronary artery	(79)
				*in vitro*	Rat H9C2 myocardial cells subjected to hypoxic treatment	
			Kaempferol ameliorates myocardial infarction injury through the HDAC3-mediated Nrf2 signaling pathway	*in vivo*	Rats induced by subcutaneous injection of ISO	(80)
				*in vitro*	Rat H9c2 cardiomyocytes stimulated with CoCl_2_	
Intracellular iron metabolism	mTOR	Overexpression of mTOR inhibits ROS and ferroptosis	Idebenone alleviates ferroptosis through the AMPK-mTOR pathway	*in vivo*	Rats with ligation of left anterior descending coronary artery	(81)
				*in vitro*	H_2_O_2_-induced Rat cardiac H9c2 cells	
	miR-214-3p	Overexpression of miR-214-3p induces cardiomyocyte ferroptosis	Inhibition of miR-214-3p or overexpression of ME2 alleviates ferroptosis	*in vivo*	Mouses with ligation of left anterior descending coronary artery	(82)
				*in vitro*	primary cultures of neonatal rat cardiomyocytes with hypoxic treatment	
Lipid peroxidation	IncRNA and circRNA	lncRNAs and circRNAs regulate ferroptosis in myocardial infarction by acting as miRNA sponges, thereby preventing miRNAs from binding to their target mRNAs	LncRNA AC005332.7 sponges miR-331-3p and regulates CCND2 to inhibit ferroptosis and alleviate acute myocardial infarction injury	*in vivo*	Mouses with ligation of left anterior descending coronary artery	(83)
				*in vitro*	AC16 cells under oxygen and glucose deprivation conditions	
			CircRNA1615 regulates LRP6 expression by sponge adsorption of miR-152-3p and thus inhibits ferroptosis in myocardial infarction	*in vivo*	Mouses with ligation of left anterior descending coronary artery	(84)
				*in vitro*	mouse cardiomyocytes HL-1 with hypoxic treatment	
	NEAT1	NEAT1 alleviates lipid peroxidation and cardiomyocyte ferroptosis	Silencing NEAT1 ameliorates myocardial ischaemic injury by inhibiting ferroptosis via the miR-450b-5p/ACSL4 pathway	*in vivo*	Mouses with ligation of left anterior descending coronary artery	(85)
				*in vitro*	mouse cardiomyocytes HL-1 with hypoxic treatment	
	USP	Inhibition of USP expression reduces iron deposition and lipid ROS, thereby improving cardiac function	USP7 inhibitors attenuate ferroptosis through the Nrf2 pathway	*in vivo*	Rats with ligation of left anterior descending coronary artery	(86)
				*in vitro*	Rat cardiomyocyte H9c2 cells with OGD/R	
			SP1 enhances USP46 transcription to Inactivate AMPK signalling and exacerbates ferroptosis after myocardial infarction	*in vitro*	cardiomyocytes AC16 with OGD/R	(87)
	HO-1	Overexpression of HO-1 induces iron overload in the endoplasmic reticulum of cardiomyocytes	HO-1 expression Is upregulated by Nrf2 through the Nrf2/Hmox1 pathway in the early and mid-term of myocardial infarction by Nrf2	*in vivo*	Fifty STEMI patients reperfused by PPCI	(88)
			FNDC5/Irisin activates the Nrf2 signalling pathway to slow down Nrf/HO-1 pathway-mediated ferroptosis	*in vitro*	Cardiomyocytes with hypoxic treatment	(89)
	m6A	m6A plays a role in cardiomyocyte ferroptosis by dynamically regulating RNA stability, splicing, transport, and translation	WTAP-mediated modification of KLF6 m6A exacerbates injury and ferroptosis in AC16 cardiomyocytes under hypoxia treatment	*in vitro*	Human cardiomyocyte (AC16) with hypoxic treatment	(90)

### 4.1 System X^c−^ and GPX4 pathways

The system X^c−^ and GPX4 pathways represent critical regulatory mechanisms of ferroptosis, exhibiting significant roles in MI. The function of system X^c−^ is mainly to promote the synthesis of GSH by exchanging Cys with Glu, thus maintaining the antioxidant capacity of cells (22). It has been shown that HO-1 modulates the activity of SLC7A11 through its expression in cardiomyocytes, subsequently influencing the function of system X^c−^. Overexpression of HO-1 upregulates SLC7A11, which in turn slows down the onset of ferroptosis, and the activation not only contributes to the reduction of cardiomyocyte injury, but also provides novel strategies for cardioprotection following MI (91). Additionally, the specific microRNA (miRNA) targets determine their properties to inhibit or promote ferroptosis. miR-432-5p promotes the activation of Nrf2 and regulates the expression of GPX4 and SLC7A11 by binding to Keap1, which enhances the antioxidant capacity of cells to suppress ferroptosis (76). These findings underscore the protective role of the Nrf2-GPX4/SLC7A11/HO-1 pathway in MI, which preserves cardiomyocyte survival by reducing iron load and lipid peroxidation. Conversely, miR-15a-5p, a direct target of GPX4, exacerbates ferroptosis and aggravates hypoxia-induced cardiomyocyte injury when overexpressed, modulating ferroptosis in acute myocardial infarction (AMI) (74).

The downregulation of GPX4 during MI contributes to ferroptosis in cardiomyocytes under metabolic stresses such as cysteine deprivation (72). Within the GPX4 pathway, curdione, a sesquiterpenoid derived from *Radix Curcumae*, has been shown to markedly attenuate isoproterenol (ISO) induced MI as evidenced by reduced MDA and iron levels alongside elevated GSH levels and GPX4 expression (17). Protein levels of GPX4 are significantly downregulated in the early and intermediate stages of MI, with cysteine deprivation induced GSH depletion further suppressing its expression, thereby enhancing the susceptibility of primary neonatal rat cardiomyocytes to ferroptosis (72). The A1 and A2b adenosine receptors have been identified to modulate GPX4, influencing ferroptosis outcomes in MI rats (92). Salvianolic acid B inhibits ferroptosis in rat MI by upregulating Nrf2 expression within the Nrf2/system X^c−^/GPX4 axis (77). Curdione disrupts the interaction between Keap1 and thioredoxin 1 (Trx1) and inhibits ferroptosis in ISO-induced MI in mice and H9C2 cells by modulating the Keap1/Trx1/GPX4 signaling pathway (17). Panaxatriol saponin (PTS) reduces angiotensin II (Ang II)-induced differentiation and proliferation of MI fibroblasts by blocking the Nrf2-binding site in Keap1, thereby inhibiting oxidative stress and destabilising the Keap1-Nrf2 interaction (78). Geniposide (GEN), the main active ingredient of *Gardenia jasminoides J. Ellis*, directly upregulates the expression of the RNA-binding protein of GPX4, G-rich RNA sequence-binding factor 1 (Grsf1), at the translational level via the Grsf1/GPX4 axis following myocardial oxidative injury. This mechanism reduces iron overload and lipid peroxidation in MI rats and thus counteracts ferroptosis injury after MI (75).

In addition, the role of Aldo-keto reductase 1C3 (AKR1C3) as a stress-regulated gene in MI is of interest. It was found that AKR1C3 in AMI rats and H9C2 cells preconditioned with hypoxia mitigates ferroptosis after MI by activating the Keap1-Nrf2-antioxidant response element (ARE) pathway (79). This regulatory mechanism may provide a potential therapeutic target for developing novel strategies against ferroptosis. Furthermore, Apelin has been shown to inhibit ferroptosis in cardiomyocytes by activating the AMP-activated protein kinase (AMPK) signaling pathway in a mouse model of MI induced by left anterior descending (LAD) coronary artery ligation and thus exerts protective effects following MI (93).

### 4.2 Intracellular iron metabolism

Homeostatic regulation of iron plays a central role in the initiation of ferroptosis. In the pathological context of MI, dysregulated iron metabolism is often accompanied by iron overload and the accumulation of ROS (39). In this process, key genes related to iron metabolism, such as mTOR, exert significant regulatory effects. mTOR prevents ferroptosis mediated damage in MI by modulating iron metabolism. Cardiac overexpression of mTOR suppresses ROS production and ferroptosis, whereas mTOR downregulation promotes ferroptosis alongside increased ROS generation (94). mTOR operates through two multiprotein complexes, the mammalian target of rapamycin complex 1 (mTORC1) and the mammalian target of rapamycin complex 2 (mTORC2), which play critical roles in stress responses (95). mTORC1 regulates the efficiency of translation initiation by targeting S6K1, 4E-BP1 as part of a complex cellular signaling network (96, 97). Idebenone, a synthetic analog of coenzyme Q10, mitigates ferroptosis by modulating excessive autophagy through the AMPK-mTOR pathway, thereby exerting cardioprotective effects in MI (81).

Nrf2, a transcription factor implicated in ferroptosis regulation, further underscores the critical role of iron metabolism in MI. Kaempferol reduces oxidative stress and ameliorates MI injury through the histone deacetylases 3 (HDAC3) mediated Nrf2 signaling pathway in cardiomyocytes (80). Hematopoietic progenitor kinase 1-interacting protein of 55 kDa (HIP-55), an adaptor protein, is the nodal regulator and hub protein in cardiomyocyte ferroptosis. Protein kinase B (Akt) phosphorylates HIP-55 at S269/T291 site, enabling HIP-55 to coordinate the dynamic interplay between Akt mediated cell survival and the mitogen-activated protein kinase kinase 1 (MAP4K1) dependent c-Jun amino-terminal kinase (JNK)/GPX4 ferroptosis pathway, thereby protecting against ferroptosis in MI (98). Heme degradation, which generates Fe^2+^, is essential for maintaining iron homeostasis. BTB domain and CNC homolog 1 (BACH1), a regulator of heme and iron metabolism, promotes ferroptosis by suppressing the transcription of a subset of protective genes induced by erastin, as demonstrated in a mouse model of AMI induced by left coronary artery ligation (99). miR-214-3p has been identified to exacerbate ferroptosis and cellular injury in neonatal rat cardiomyocytes, whereas miR-214-3p inhibitors effectively protect cells against hypoxia-induced damage. Malic enzyme 2 (ME2) is a direct target of miR-214-3p, and its overexpression effectively mitigates excessive ferroptosis induced by miR-214-3p mimics. miR-214-3p induces ferroptosis in MI by targeting ME2 (82). Precise regulation of iron metabolism represents a promising direction for reducing ferroptosis and improving outcomes in MI. Epigallocatechin gallate, the primary polyphenol in green tea, reduces AMI injury by preventing ferroptosis through the miR-450b-5p/ACSL4 axis (14).

### 4.3 Lipid peroxidation

Lipid peroxidation plays a central role in the mechanisms underlying ferroptosis, particularly in the context of MI. Lipid peroxidation triggered by iron overload is recognized as a critical factor driving ferroptosis. Bulluck et al. (100) demonstrated that residual myocardial iron might be a potential therapeutic target for reducing adverse ventricular remodelling in patients with reperfused MI. Lipid metabolism is essential for maintaining optimal cardiovascular function. The occurrence of ferroptosis is closely related to lipid peroxidation, especially the oxidation of PUFA in cell membranes, which disrupts membrane structure and function, which in turn triggers ferroptosis (101). Dysregulation of the system X^c−^ and GPX4 pathways leads to the accumulation of lipid ROS, exacerbating membrane damage and promoting ferroptotic cell death.

The modulation of lipid peroxidation has emerged as a critical research focus in MI studies. The suppression of lipid peroxidation has been demonstrated to effectively attenuate ferroptosis progression, thereby reducing the damage after MI. Noncoding RNAs are pivotal in the intricate interplay between MI and ferroptosis (102). Long-stranded noncoding RNAs (IncRNAs) mediate physiological and pathological processes in diseases such as AMI. For instance, IncRNA AC005332.7 alleviates ferroptosis and mitigates AMI injury by sponging miR-331-3p, a biomarker in ST-segment elevation myocardial infarction (STEMI), and regulating crucial roles of cyclin D2 (CCND2) expression (83). CircRNA1615 inhibits ferroptosis in MI through sponge adsorption of miR-152-3p, which modulates the expression of low-density lipoprotein receptor-related protein 6 (LRP6) (84). Nuclear paraspeckle assembly transcript 1 (NEAT1) has been shown to reduce lipid peroxidation and ferroptosis in hypoxic HL-1 cells and AMI mice. NEAT1 directly sponges miR-450b-5p and negatively regulates its expression. miR-450b-5p directly targets ACSL4 and the silencing of NEAT1 suppresses ferroptosis via the miR-450b-5p/ACSL4 axis, effectively ameliorating myocardial ischemic injury (85).

Ubiquitin-specific protease (USP), the largest subgroup of deubiquitinating enzymes, plays a pivotal role in the progression of CVDs (103). Nrf2, identified as a master transcriptional regulator in ferroptosis, exerts critical functions within the Nrf2-lipid peroxidation-ferroptosis axis by protecting cells from lethal ROS damage, modulating antioxidant responses across diverse cell types and suppressing ferroptosis (52). Studies have revealed that USP7 is highly expressed in ferroptosis-mediated MI. Inhibition of USP7 activates the Keap1-Nrf2 pathway, elevates nuclear Nrf2 expression, reduces iron deposition and lipid ROS levels, and improves cardiac function while diminishing infarct size through ferroptosis attenuation (86). Specificity protein 1 (SP1) mediates ferroptosis induced by ischemia-reperfusion injury after MI by enhancing the transcription of USP46 and inactivating AMPK signaling (87). Iron overload in the endoplasmic reticulum of cardiomyocytes is triggered by HO-1 overexpression under hypoxic or hypoxia/reoxygenation conditions, leading to ferroptosis via iron metabolic pathways (104). Furthermore, HO-1 expression is upregulated by Nrf2 through the Nrf2/heme oxygenase 1 (Hmox1) pathway during the early and intermediate stages of MI, resulting in iron excess and subsequent ferroptosis of cardiomyocytes (88). Irisin, a myokine derived from proteolytic cleavage of the extracellular domain of fibronectin type III domain-containing 5 (FNDC5), is a transmembrane protein that enhances cardiomyocyte viability and reduces oxidative stress. FNDC5/Irisin expression is downregulated in hypoxic cardiomyocytes, whereas its activation of the Nrf2 signaling pathway mitigates ferroptosis through the Nrf2/HO-1 axis (89). Adipsin, an adipokine, significantly upregulates ferritin heavy chain (FtH) levels while downregulating transferrin receptor (TFRC) expression and alleviating lipid oxidative stress associated with MI (105). Differentially expressed genes (DEGs) identified via microarray analysis in AMI are linked to m6A modifications and ferroptosis, which offer novel insights for timely diagnosis and treatment (106). WT1-associated protein (WTAP) mediated m6A modification of kruppel-like factor 6 (KLF6) exacerbates hypoxic injury and ferroptosis in AC16 cardiomyocytes under low-oxygen conditions (90).

## 5 Discussion

Ferroptosis is an emerging form of cell death whose mechanism involves multiple biological processes in which the regulation of protein translation plays a crucial role. Traditionally, ferroptosis has been primarily associated with iron metabolism and lipid peroxidation (61). However, recent studies have revealed that dysregulation of protein translation, particularly abnormalities in translation initiation, elongation, and termination, may significantly influence the initiation and progression of ferroptosis (54–57). The occurrence of ferroptosis is closely linked to the system X^c−^ and GPX4 pathways, where system X^c−^ facilitates Cys transport, thereby promoting GSH synthesis (25, 26). GSH is a key substrate for GPX4, inhibiting lipid peroxidation through its antioxidant activity and preventing ferroptosis (29). Research has demonstrated that translation initiation factors (e.g., eIF2α) are non-negligible in ferroptosis. Phosphorylation of eIF2α modulates the translation initiation rate and regulates ferroptosis by influencing cellular oxidative stress responses (107, 108). Furthermore, the accumulation of lipid peroxides represents a central factor in ferroptosis, while translation factors involved in protein synthesis are intricately linked to regulating cellular stress responses and potentially modulating ferroptosis sensitivity through the expression of antioxidant genes (53).

Although extensive studies have elucidated the role of protein translation in ferroptosis, current research still faces several limitations. (i) The precise regulatory mechanisms of ferroptosis remain incompletely understood, particularly the specific interactions between intracellular iron metabolism and protein translation. While certain translation factors and post-translational modifications have been identified as closely associated with ferroptosis, their molecular-level coordination and collective regulation of ferroptosis require further investigation. More importantly, ferroptosis is not an isolated event but is intricately linked to other forms of cell death, such as apoptosis, necrosis, and autophagy. The molecular mechanisms governing this crosstalk need to be further characterized to uncover potential antagonistic or synergistic effects in the context of CVDs, particularly MI. (ii) Most of the research on MI predominantly relies on animal models and cell-based experiments, with a lack of extensive clinical validation. Consequently, translating these fundamental findings into clinical therapeutic strategies remains a significant challenge. (iii) The feedback mechanisms between protein translation and ferroptosis have not been thoroughly explored. Although regulatory mechanisms such as the phosphorylation of translation initiation factors play a role in ferroptosis, their functions may vary across different cell types and disease contexts. Therefore, exploring the impact of translational regulation on ferroptosis in diverse disease settings, particularly in complex conditions such as MI, represents a critical direction for future research.

Future research could elucidate the intricate relationship between protein translation and ferroptosis, with particular emphasis on their specific roles in MI. (i) Studies should prioritize the details of how translation factors are involved in the regulation of ferroptosis, revealing how various aspects of the protein translation process interact with iron metabolism and antioxidant systems, etc. (ii) As understanding of PTMs advances, researchers are encouraged to explore therapeutic strategies that target specific PTM-modifying enzymes or associated molecules to modulate ferroptosis, potentially offering novel interventions for MI and related pathologies. (iii) Clinical investigations in this field must be prioritized. While current ferroptosis research predominantly focuses on preclinical models, translating these findings into clinical applications holds significant promise. For instance, developing miRNA-based therapies, protein-specific modulators, or small-molecule compounds capable of regulating protein translation pathways to attenuate or reverse myocardial injury post-MI represents an emerging frontier in clinical research. Building on the interplay between protein translation and ferroptosis, the design of innovative therapeutic approaches targeting MI may provide patients with additional treatment modalities.

## 6 Conclusions

This review provides an exhaustive exploration of the relationship between ferroptosis and protein translation, elucidating their potential mechanistic roles in MI. By synthesizing the significant themes and findings from the preceding five years of research, novel strategies for preventing and treating MI are proposed. Based on a comprehensive analysis of ferroptosis and protein translation mechanisms, targeting translation factors or modulating key proteins involved in iron metabolism and lipid peroxidation may offer potential therapeutic avenues to inhibit ferroptosis, thereby mitigating cardiomyocyte injury and death. Future research should focus on the intersecting regulatory networks between translation mechanisms and ferroptosis to identify innovative therapeutic strategies for MI and enhance treatment efficacy.
